# Remnant cholesterol has an important impact on increased carotid intima-media thickness in non-diabetic individuals

**DOI:** 10.1007/s10554-023-02957-0

**Published:** 2023-09-28

**Authors:** Xiaoqiong Du, Jie Ding, Xinchen Ma, Ruijie Yang, Luna Wang, Dujuan Sha

**Affiliations:** 1 Nanjing Drum Tower Hospital Clinical College of Nanjing Medical University, Nanjing, Jiangsu 211166 China; 2 Department of Health Management Center, Affiliated Drum Tower Hospital of Nanjing University Medical School, Nanjing, China; 3 Department of General Practice, Affiliated Drum Tower Hospital of Nanjing University Medical School, Nanjing, China

**Keywords:** Remnant cholesterol, Carotid intima-media thickness, Non-diabetic individuals

## Abstract

To investigate the correlation the correlation between residual cholesterol (RC) and increased carotid intima-media thickness(cIMT) in non-diabetic individuals. This study included 1786 non-diabetic individuals who underwent carotid ultrasound. RC was calculated based on total cholesterol (TC), LDL-C, and high density lipoprotein cholesterol (HDL-C). The subjects were divided into the cIMT thickening group (cIMT ≥ 0.1 cm) and non-thickening group (cIMT < 0.1 cm) groups based on cIMT, binary logistic regression with different models and receiver operating characteristic (ROC) curves were adopted to evaluate the predictive ability of RC in cIMT. Of the research participants , their median age was 55 (49–51) years, 1121 (63%) were male, and 209 (12%) had hypertension, and people in the cIMT thickening group (925) were more likely to be older and male than those in the non-thickening group (843). Across the different RC subgroups, there was an increasing trend in maximum cIMT (*P* < 0.001) as RC levels increased within quartiles. RC was found to be an independent risk predictor for cIMT thickening (all *P* <  in models 1–3); and this result persisted in the LDL-C normal subgroup (*P* = 0.002). The results suggested that RC was an independent predictor of cIMT thickening in non-diabetic individuals and had a strong atherogenic effect.

## Introduction

LDL-C is known to be a major contributor to AS and the target of current treatment and primary prevention strategies for atherosclerotic cardiovascular disease (ASCVD) [1, 2]. Analyses have shown that individuals receiving statins have a significant residual cardiovascular risk, even those with normal LDL-C levels [3]; however, some studies have suggested that adding medication targeting triglycerides (TG) and remnant cholesterol (RC) to statin therapy may be more effective [3–5]. Recent epidemiological studies have shown that elevated RC, an independent factor in the development of cardiovascular events, is associated with ASCVD risk. RC has better ability to predict cardiovascular disease risk compared to LDL-C [6–11].

RC is the cholesterol content of TG-rich lipoproteins, including intermediate-density lipoproteins (IDLs), very low-density lipoproteins (VLDLs), and remnants of celiac particles in the non-fasting state. Quispe et al. [6] reported that residual particles have similar atherogenic capacity to LDLs but carry 40 times more cholesterol than VLDLs and can pass through the endothelium. The unique physicochemical characteristics of remnant particles make them highly atherogenic and important risk factors of cardiovascular events.

Carotid arteries are a high−prevalence site for atherosclerosis, and increased cIMT and carotid plaques, especially unstable atherosclerotic plaques, are high-risk factors for ischemic stroke [12, 13]. cIMT is a relatively simple measure of large artery atherosclerosis and is widely used to assess cardiovascular events due to its noninvasive and convenient nature [14]. Many researchers have analyzed conventional lipid parameters such as LDL-C or TG in relation to cIMT thickening [15, 16]; however, there are few reports on the association between RC and cIMT thickening. Therefore, this work was performed to evaluate the predictive ability of RC in cIMT thickening in a non-diabetic population.

## Methods

### Study subjects

**Fig. 1:**
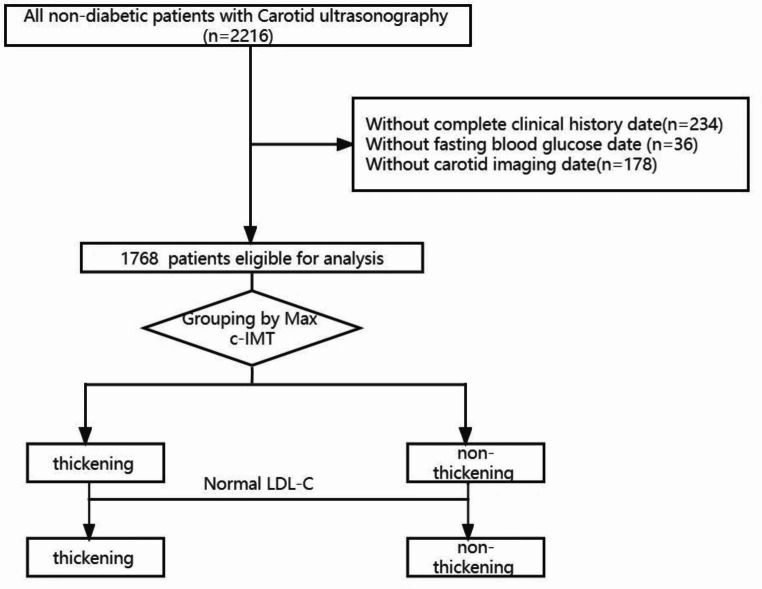
A study flowchart of enrollment

A amount of 2216 non-diabetic participants who underwent carotid ultrasound from January 2018 to October 2021 at the Drum Tower Hospital Affiliated with Nanjing University (Nanjing, Jiangsu,China) were initially enrolled in this research. All the study participants were screened, and patients were excluded if they were aged > 80 or < 18, had cancer or severe chronic illness, or had incomplete data. Patients with an incomplete clinical history (n = 234), missing fasting glucose levels (n = 36), and incomplete carotid ultrasound values (n = 178) were also excluded (Fig. 1). This study was approved by the Ethics Committee of Drum Tower Hospital Affiliated to Nanjing University (ethics code 2022-333-01, approval date 01-May-2022), all participants signed an informed consent to participate in the study.

### Data collection and measurement

Basic clinical data were retrospectively collected from the non-diabetic population as follows: (1) demographic characteristics: sex and age; (2) past history: hypertension, coronary heart disease (CAD), stroke; (3) laboratory tests: red blood cell distribution width (RDW), fasting glucose, TC, TG, LDL-C and HDL-C; fasting glucose, TC, TG, LDL-C, and HDL-C levels were assayed with an automated biochemical analyzer, while RDW was measured with an automated hematology analyzer. There is no standard assay for RC; thus, a formula commonly used in previous studies was used to calculate RC values (RC = TC-LDL-HDL) [17, 18]. The atherosclerosis index (AI), a non-conventional lipid parameter, was also calculated (AI = non-HDLC/HDL-C).

### Carotid intima-media thickness measurement

**Fig. 2:**
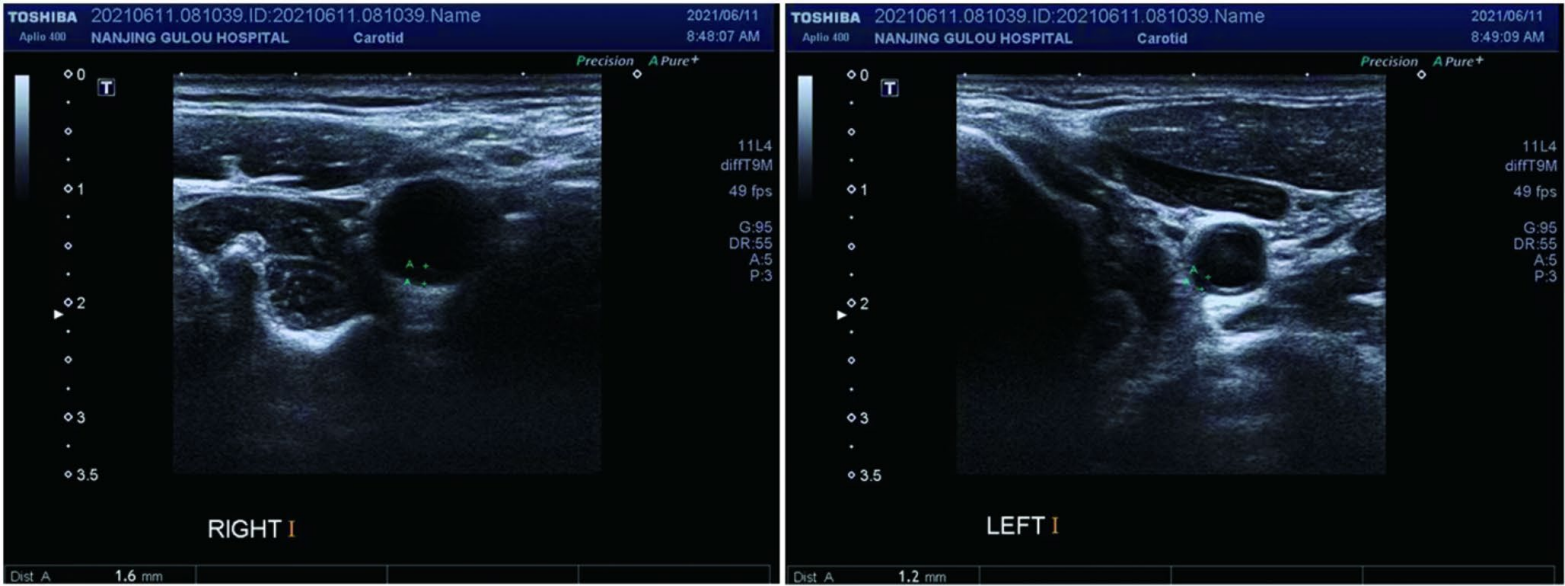
Measurement of carotid intima-media thickness

All participants underwent carotid Doppler scanning(Philips HD5G, GE, USA, 5 ~ 12 MHz), in which the ultrasound probe was moved cephalad from the root of the neck in a transverse sweep, assessing the distal common carotid artery, the carotid bifurcation, and the proximal internal carotid artery, respectively. Multi-point and multiple measurements were performed on both sides of the carotid intima, and the maximum and average values of the left and right sides were calculated for subsequent analysis (Fig. 2). Two qualified physicians in the ultrasound department performed the carotid ultrasound examinations and reported the results. cIMT ≥ 1 mm was defined as thickening [19].

### Other covariates

Hypertension was defined as systolic blood pressure (BP) ≥ 140 mmHg or diastolic BP ≥ 90 measured three times on different days, self-reported physician diagnosis or taking blood pressure-lowering medication [20]. Diabetes was diagnosed as a fasting blood glucose ≥ 11.1 mmol/l or non-fasting blood glucose ≥ 7.0 mmol/l, a reported history of diabetes or taking glucose-lowering medication [21]. CAD referred to a heart disease caused by ischemia and hypoxia of the myocardium due to atherosclerosis of the coronary arteries [22]. It is generally believed that ischemic stroke is caused by severe narrowing or occlusion of cerebral blood vessels due to impaired cerebral blood circulation and consequent ischemia and hypoxia leading to brain tissue necrosis in the blood supply area, often confirmed by cranial CT or MRI images [23].

### Statistical analysis

The enrolled population was grouped according to the cIMT as well as RC quartiles, respectively. The measurement data were expressed as the mean ± standard deviation (x̄ ± S), and the count data were expressed as frequency and percentage (%); independent samples t-tests or chi-square (χ2) tests were used for comparison between the two groups. One-way ANOVA was adopt for comparisons between multiple groups; non-normally distributed measures were expressed in terms of medians and quartiles, and a non-parametric U test was used. The Spearman correlation between RC and each variable was determined, and the relationship between cIMT and each variable was analyzed by binary logistic regression. Multi-model logistic regression was used to analyze the predictive ability of RC for cIMT thickening and to calculate intergroup trends after correction for confounders. The patients with normal LDL-C levels were screened and grouped according to RC quartiles, and the subgroups were evaluated using ANOVA to explore whether the observed correlation was independent of LDL-C.

## Results

### Baseline characteristics of participants according to cIMT

**Fig. 3:**
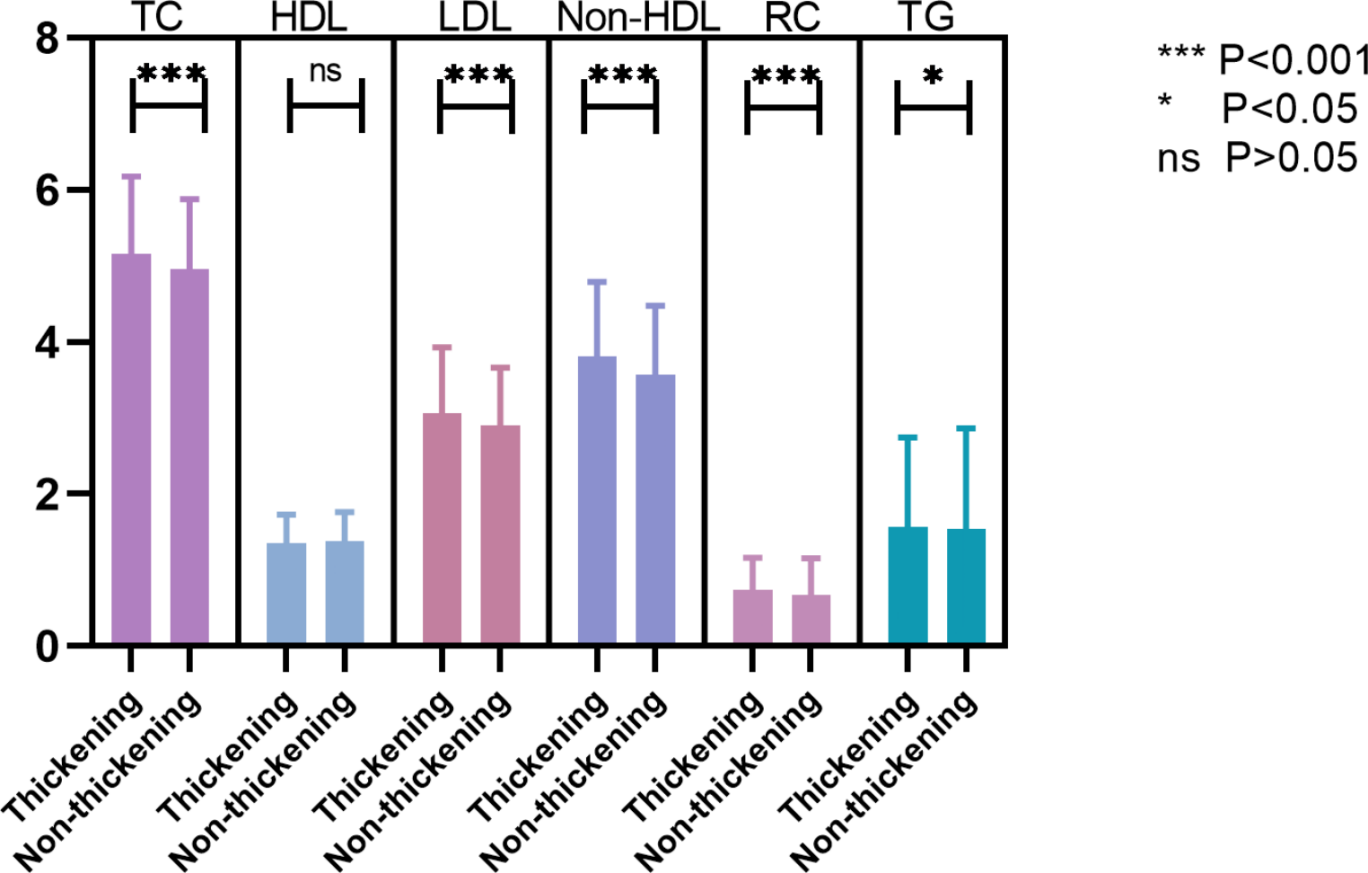
Lipid concentration distribution among different groups. According to the maximum carotid intima-media thickness, the patients were divided into two groups. Mann-whitney U test was used to analyze the results

Among the 2216 non-diabetic individuals who underwent carotid ultrasound, 1786 were selected in the ultimate analysis. Of these individuals, 925 in the cIMT thickening group and 843 in the non-thickening group. Figure 1 showed the specific screening process, while Table 1 depicted the baseline characteristics of the subjects. Of the research participants, their median age was 55 (49–51) years, 1121 (63%) were male, and 209 (12%) had hypertension. Those with cIMT ≥ 0.1 cm were in the cIMT thickening group, and those with patients had higher fasting glucose, systolic BP, LDL-C, TC, TG, non-HDL, and RC levels and lower HDL-C levels (Table 1; Fig. 3).

**Table 1:** Baseline characteristics of participants According to cIMT

Variables	cIMT thickening (925)	cIMT non-thickening (843)	*P*
Age (years) Male gender, n (%) Hypertension, n (%) Hyperlipidemia, n(%) Stroke, n(%) RDW, % Fasting glucose, mmol/L Systolic BP,mmHg Diastolic BP,mmHg LDL-C, mmol/l HDL-C, mmol/l Total cholesterol, mmol/l RC, mmol/l Triglycerides, mmol/l Non-HDL-C, mmol/l AI	58(53–67) 621(67) 141(15) 19(2) 55(6) 12.9(12.6–13.2) 5.21(4.87–5.69) 136(123–149) 83(75–90) 3.07(2.5–3.63) 1.29(1.07–1.58) 5.13(4.51–5.78) 0.67(0.52–0.86) 1.29(0.95–1.87) 3.8(3.2–4.41) 2.91(2.22–3.72)	52(52–57) 500(59) 68(8) 7(0.8) 24(3) 12.7(12.4–13.1) 4.9(4.5–5.3) 125(115–137) 79(71–87) 2.87(2.4–3.39) 1.33(1.1–1.6) 4.88(4.31–5.56) 0.59(0.42–0.8) 1.22(0.86–1.79) 3.53(2.95–4.14) 2.65(1.98–3.53)	< 0.001 0.001 < 0.001 0.033 < 0.001 < 0.001 < 0.001 < 0.001 < 0.001 < 0.001 < 0.001 0.08 < 0.001 0.012 < 0.001 < 0.001

Abbreviations: RDW red cell distribution width,BP blood pressure,HDL-C high-density lipoprotein cholesterol,LDL-C low-density lipoprotein cholesterol,RC remnant cholesterol,AI atherosclerosis index

### Baseline characteristics of participants according to RC quartiles

The 1786 non-diabetic individuals were divided by RC quartiles, 427 in group Q1, 437 in group Q2, 456 in group Q3, and 443 in group Q4. The comparison of the baseline characteristics among the groups showed that patients with higher RC levels were more possible to be older and male than those with lower RC levels; those with high RC levels also had higher LDL-C, TC, TG, non-HDL-C, fasting glucose, and BP levels. The prevalence of hypertension, CAD, and stroke was mostly balanced among the four groups. There was an increasing trend in maximum cIMT (*P* < 0.001) as RC levels increased within quartiles (Table 2).

**Table 2:** Baseline Characteristics According to RC Quartiles

Variables	<0.47(427)	0.47–0.63(437)	0.63–0.83(456)	≥ 0.83(443)	*P*
Age (years) Male gender, n (%) Hypertension, n (%) Hyperlipidemia, n(%) Stroke, n(%) RDW, % Fasting glucose, mmol/L Systolic BP,mmHg Diastolic BP,mmHg LDL-C, mmol/l HDL-C, mmol/l Total cholesterol, mmol/l Triglycerides, mmol/l Non-HDL-C, mmol/l AI Max-cIMT(mm) Mean cIMT(mm)	44(54–61) 212(49.6) 47(11) 7(1.6) 15(3.5) 12.4(12.7–13.1) 4.52(4.87–5.31) 113(125–141) 70(77–87) 2.18(2.6–3.11) 1.35(1.58–1.89) 4.09(4.56–5.16) 0.6(0.77–0.94) 2.53(2.96–3.5) 1.45(1.81–2.31) 0.07(0.09–0.11) 0.07(0.08–0.105)	50(57–65) 271(62) 60(13.7) 8(1.8) 25(5.7) 12.5(12.7–13.1) 4.71(5.03–5.46) 118(131–144) 72(80–88) 2.48(2.92–3.45) 1.19(1.39–1.62) 4.33(4.87–5.55) 0.9(1.08–1.27) 3.04(3.48–3.99) 2.035(2.52–2.98) 0.08(0.1–0.12) 0.07(0.095–0.105)	50(55–62) 296(64.9) 45(9.9) 8(1.8) 15(3.3) 12.5(12.8–13.2) 4.71(5.06–5.49) 121(132–143) 75(81–89) 2.66(3.18–3.62) 1.07(1.24–1.47) 4.59(5.18–5.69) 1.21(1.48–1.77) 3.37(3.88–4.34) 2.5(3.07–3.64) 0.08(0.1–0.12) 0.07(0.095–0.105)	49(55–60) 338(76.3) 56(12.6) 3(0.7) 24(5.4) 12.5(12.9–13.2) 4.88(5.19–5.78) 123(135–149) 77(84–93) 2.54(3.16–3.78) 0.93(1.07–1.27) 4.83(5.39–6.12) 1.88(2.33–3.15) 3.74(4.35–4.95) 3.4(3.98–4.62) 0.08(0.1–0.12) 0.07(0.095–0.105)	0.002 <0.001 0.289 0.450 0.177 0.015 < 0.001 < 0.001 < 0.001 < 0.001 < 0.001 < 0.001 < 0.001 < 0.001 < 0.001 < 0.001 < 0.001

**Abbreviations**: RDW red cell distribution width, BP blood pressure, HDL-C high-density lipoprotein cholesterol, LDL-C low-density lipoprotein cholesterol, RC remnant cholesterol, AI atherosclerosis index, cIMT carotid intima-media thicknes

### Correlation analysis and logistic regression

**Fig. 4:**
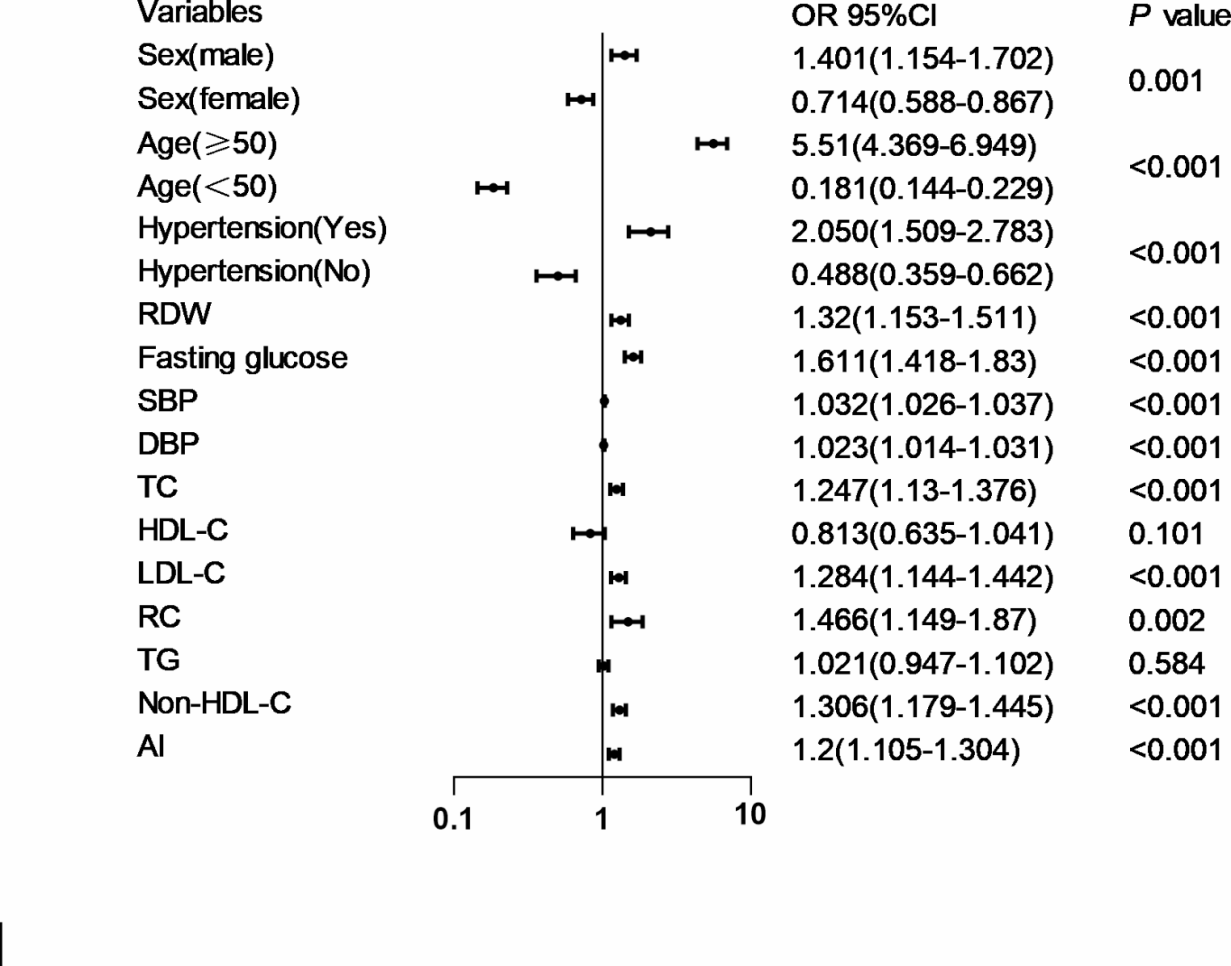
Logistic regression analysis evaluating predictive implication of RC in various stratifications. Abbreviations: HDL-C high-density lipoprotein cholesterol,LDL-C low-density lipoprotein cholesterol,RC remnant cholesterol,TC total cholesterol,TG triglycerides,BP blood pressure,AI atherosclerosis index,CI=confidence interval

The Spearman’s correlation analysis showed that RC was positively correlated with age,fasting glucose, RDW, BP parameters, TG, TC, LDL-C, non-HDL-C, and AI levels and negatively correlated to HDL-C levels (Table 3). Intergroup trends in RC quartiles were assessed by calculating the median within each quartile. Three models (models 1−3) were developed, including statistically significant covariates and clinically significant models to evaluate the predictive ability of RC in cIMT thickening. cIMT increased over the interquartile range of RC as the variables in the models were continuously adjusted. In model 1, the OR for the highest quartile of RC was 2.175 (95% CI = 1.593−2.969, *P* < 0.001) compared with the lowest quartile. The adjustment of model 2 did not change anything significantly. In model 3, with further adjustment for statistically significant variables and exclusion of confounders affecting RC, the corresponding ORs for the highest and lowest quartiles of RC were 2.376 (95% CI = 1.508−3.745) and 1.658 (95% CI = 1.18−2.33). Additionally, RC was entered as a categorical variable into the model for analysis, and *P*-values were obtained for the 3 comparisons. Larger RC values were an independent risk predictor for cIMT (*P* < 0.001) (Table 4). Further research was applied to explore the correlation between cIMT and each variable; the results revealed RC level (OR = 1.466, CI = 1.149−1.87, *P* = 0.002) to be an independent risk factor in cIMT thickening (Fig. 4).

**Table 3:** Correlations between the RC and other variables

Variables	Correlation coefficient	*P* value
Age Max-cIMT Mean cIMT RDW Fasting glucose Systolic BP Diastolic BP LDL-C HDL-C Total cholesterol Triglycerides Non-HDL-C AI	0.03 0.119 0.116 0.073 0.213 0.166 0.217 0.245 -0.555 0.329 0.833 0.562 0.743	0.211 <0.001 <0.001 0.002 <0.001 <0.001 <0.001 <0.001 <0.001 <0.001 <0.001 <0.001 <0.001

The Spearman correlation analysis was used to determine the correlations between RC and other continuous variables, including age, fasting glucose, total cholesterol, triglycerides, RDW, Max-cIMT, Mean-cIMT, systolic BP, diastolic BP, LDL-C, HDL-C, Non-HDL-C, AI

**Abbreviations**: cIMT carotid intima-media thickness,RDW red cell distribution width, BP blood pressure, HDL-C high-density lipoprotein cholesterol, LDL-C low-density lipoprotein cholesterol, AI atherosclerosis index

**Table 4:** Logistic models to assess the predictive power of RC on AS

	**OR** (95%CI)				*P* value for Trend
	**Q1** **(n = 427)**	Q2 (n = 437)	Q3 (n = 456)	Q4 (n = 443)	
Model 1	1.0	1.581(1.156–2.16)	2.249(1.651–3.062)	2.175(1.593–2.969)	<0.001
p Value		0.004	<0.001	<0.001	
Model 2	1.0	1.567(1.146–2.144)	2.244(1.647–3.056)	2.154(1.577–2.943)	<0.001
p Value		0.005	<0.001	<0.001	
Model 3	1.0	1.658(1.18–2.33)	2.376(1.647–3.427)	2.376(1.508–3.745)	<0.001
p Value		0.004	<0.001	<0.001	

Model 1: adjusted for age, sex

Model 2: further adjusted for history of hypertension, history of hyperlipidemia, history of stroke

Model 3: further adjusted for Non-HDL-C, AI, Fasting glucose, Systolic BP, Triglyceride and Total cholesterol

**Abbreviations**: BP blood pressure, HDL-C high-density lipoprotein cholesterol, RC remnant cholesterol, AI atherosclerosis index

### Analysis after LDL-C adjustment

Notably, RC was still related to cIMT thickening after adjustment for LDL-C values. People with normal levels of LDL-C were selected into the subgroup analysis and grouped by RC quartiles to analyze the differences in cIMT among these groups. The RC quartiles were calculated as 0.43, 0.58, and 0.78 mmol/l, respectively, and evaluated using ANOVA. The distribution of Max-cIMT was significantly different between the four groups (F = 4.805, *P* = 0.002). Tukey’s test showed that the mean Max-cIMT score increased by 0.006 and 0.008 (95% CI: 0.0002−0.012, 0.0024−0.014) from group Q1 to group Q3, respectively, and the difference was statistically significant (*P* = 0.04, *P* = 0.002; Table 5).

**Table 5:** The association between RC value and c-IMT under normal LDL-C dates

Variables	‾X ± S				*F*	*P*
	**Q1** **(n = 247)**	**Q2** **(n = 254)**	**Q3** **(n = 252)**	**Q4** **(n = 252)**		
Max cIMT(mm)	**0.088 ± 0.02**	**0.094 ± 0.02**	**0.097 ± 0.03**	**0.094 ± 0.02**	**4.805**	**0.002**
Mean cIMT(mm)	**0.084 ± 0.02**	**0.089 ± 0.02**	**0.09 ± 0.02**	**0.088 ± 0.02**	**4.274**	**0.005**

ROC Curves.

Figure 5 showed the comparisons of RC and other common lipid parameters predicting cIMT thickening (cIMT ≥ 1.0 mm). The ROC results showed the AUC of RC is the largest (AUC = 0.586), revealing that RC has better ability to predict cIMT compared to conventional lipid parameters such as LDL-C or TG.

**Figure:**
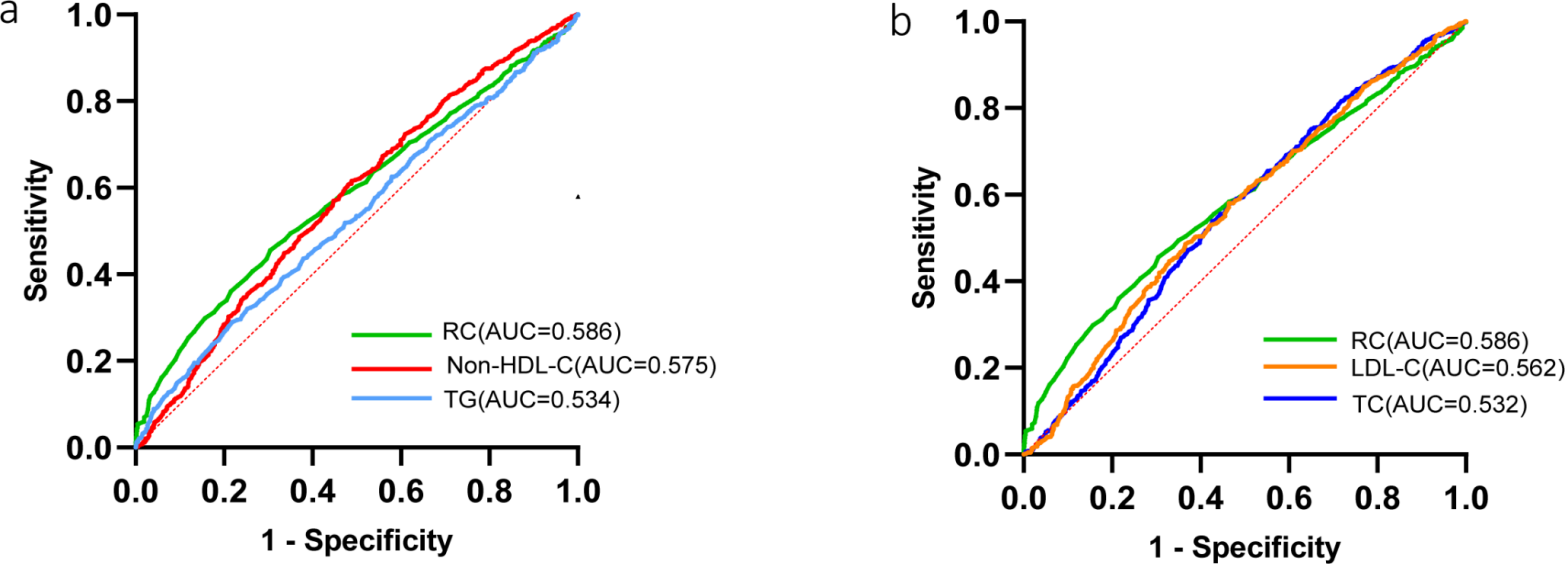
Fig. 5 ROC curve evaluating predictive effect of RC and other non-conventional lipid parameters for cIMT thickening. (a) RC versus Non-HDL-C or TG; (b) RC versus LDL-C or TC. Abbreviations: HDL-C high-density lipoprotein cholesterol,LDL-C low-density lipoprotein cholesterol,RC remnant cholesterol,TC total cholesterol,TG triglycerides

## Discussion

This retrospective study of 1786 non-diabetic participants explored RC’s predictive ability in cIMT thickening. RC correlated with cIMT, a subclinical marker of AS. Higher RC levels were found to have stronger predictive power for cIMT thickening; additionally, RC was related with many risk factors for cardiovascular diseases. This correlation persisted in subgroups with normal LDL-C levels. The findings suggest that RC is a good predictor of cIMT thickening events in non-diabetic populations.

Numerous studies have shown that AS is a causative factor and an important predictor of ASCVD events [24, 25]. Percutaneous angiography is the currently accepted standard for the diagnosis of AS, but it is not suitable for clinical application because of its invasiveness and high cost; therefore, we recommend cIMT measurement as a more noninvasive and convenient index for AS evaluation. cIMT is a widely used subclinical indicator of atherosclerosis worldwide and can be measured simply, non-invasively, and reproducibly by ultrasound. Several studies have revealed cIMT measurements to be of great value for assessing the risk of cardiovascular disease events; thus, cIMT was chosen in this study [26–28].

The present study were in agreement with the previous researches that have shown RC to be independently connected with cardiovascular events and a significant predictor of ASCVD events [29–31]. An analysis of the PREDIMED trial indicated that TG and RC (but not LDL-C) levels were independently related to cardiovascular events in a Mediterranean population with a high prevalence of diabetes and obesity [3]. The Copenhagen studies reported an relationship between RC levels and an increased risk of ischemic stroke, with atherosclerosis as the main presumed mechanism [10]. Similarly, Lin et al. [32] found a obvious correlation between RC levels and total coronary atherosclerotic burden, which persisted in normal LDL levels, independent of HDL-C and traditional ASCVD risk factors. The study provides stronger evidence for the atherogenicity of RC by analyzing the relationship between RC and CIMT, which is subclinical evidence of atherosclerosis.

RC, the cholesterol content of all TG-rich lipoproteins, consists of VLDLs, IDLs, and chylomicron residues in the non-fasting state. Some TG-rich lipoproteins are cholesterol-rich in circulation due to delayed metabolism and are highly atherosclerotic [3, 33]. The exact mechanism by which RC causes atherosclerosis is unknown, and several potential mechanisms exist. One of the possible explanations is that residual lipoproteins can readily penetrate the arterial wall [34, 35], which is necessary for atherosclerosis to occur. Remnant lipoproteins are readily retained after entering the arterial intima and can be absorbed by macrophages without oxidative metabolism [36]. This accumulation could enhance plaque formation. Another possible explanation is that elevated RC is related to inflammation and is another mechanism that promotes atherosclerosis. One study revealed increased expression of integrins on the surface of pro-inflammatory monocytes in participants with elevated RC levels, which induces foam cell production and promotes atherosclerosis [37]. Nordestgaard et al. [35] found 37% increase C-reactive protein per 1 mmol/l increase in RC levels and a causal association with 28% higher levels, suggesting a causal relationship between elevated RC and low-grade inflammation. In addition, remnant lipoproteins are hydrolyzed by lipoprotein lipase, and their hydrolysis products induce the formation of cytokines (TNF-α, IL-6, IL-8) and cell adhesion molecules; the upregulation of pro-inflammatory factors causes a cascade of inflammatory responses. RC could also activate some relevant cellular pathways. Some remnant lipoproteins upregulate the expression of ICAM-1, TF, and VCAM-1 (preatherogenic thrombogenic molecules) in vascular endothelial cells through a redox mechanism [38] and induce the formation of superoxide, leading to endothelial cell apoptosis [39]. All these factors play major roles in atherogenesis. TG-rich lipoproteins are believed to activate platelets and coagulation pathways and support the assembly of coagulation complexes, amplifying the coagulation cascade and, ultimately, dysregulating coagulation and impairing fibrinolysis, predisposing patients to atherosclerotic embolism [40].

LDL-C is the most frequently analyzed lipid parameter and is currently the main clinical target for treating patients in danger of dyslipidemia and cardiovascular events. However, a significant residual cardiovascular risk exists in individuals receiving statins, even those with optimal LDL-C levels [3]. The study are associated with the aforementioned observation. Würtz et al. [41] found that statin treatment is relevant to significant reductions in IDL and VLDL particle concentrations and that lowering RC or TG was more beneficial in patients eligible for statin treatment [42]. Meanwhile, some studies have shown that strengthening lipid-lowering treatment for patients with high RC levels can significantly lower the incidence of ASCVD events [43]; thus, RC is a potential future treatment target. Large randomized clinical trials are still lacking, and future studies require substantial clinical data to show that treatments that lower RC levels are more effective.

The research has several differences from other studies. First, previous studies usually pay attention to the relationships between RC and cardiovascular events, while the scope of present study was narrowed to focus on the predictive ability of RC in cIMT thickening. Second, while many studies have explored the risk of cardiovascular events with conventional lipid markers such as LDL-C or TG, this study focused on RC, a non-conventional assessment. Finally, this study was conducted in a non-diabetic population, eliminating major confounders and improving the reliability of the results. This work better explained the main cause of cardiovascular disease, namely the atherogenic effect of RC, which has been shown in numerous studies to be an important risk factor for ASCVD events.

This retrospective study provides strong evidence for the correlation between RC and cIMT thickening in the non-diabetic individuals. However, limitations should also be noted. First, the subjects in this research were few, and more data are still being collected. Second, as a retrospective study, the sample included in the analysis was non-randomized, which impacts on the wider application of our results. Third, the team needs to explore the relationship between RC and cIMT thickening through prospective studies with larger samples. Finally, the use of lipid-lowering medications in the study population was not considered, which may impact the results.

There is no standard testing methods for RC, and it is recognized to use lipid calculations to obtain residual cholesterol values at no additional cost, which is expected to be widely used in clinical practice in the future. We report a new relationship between RC and increased carotid intima-media thickness in non-diabetic individuals. This population-based finding provides a better explanation for remnant cholesterol-induced cIMT thickening, that is, RC has a strong atherogenic capacity, while our study has clinical implications in exploring ASCVD events.

## Conclusions

The results suggested that RC was an independent predictor of cIMT thickening in non-diabetic individuals and had a strong atherogenic effect. This result expanded the field of assessing cIMT indicators, and highlighted RC as a new potential therapeutic target. In clinical practice, patients with normal conventional lipid indices could be monitored for RC, and targeted therapy for patients with high RC could prevent cardiovascular events; however, the findings need to be confirmed in prospective studies and clinical trials. It is expected that RC is included in routine lipid screens in the future and that targeted treatments will be developed for future clinical applications.

## Abbreviations

AI: Atherosclerosis index
AS: Atherosclerosis
ASCVD: Atherosclerotic cardiovascular disease
AUC: Area under the curve
BP: Blood pressure
CAD: Coronary heart disease
cIMT: Carotid intima-media thickness
HDL-C: High-density lipoprotein cholesterol
IDLs: Intermediate-density lipoproteins
LDL-C: Low-density lipoprotein cholesterol
RC: Remnant cholesterol
RDW: Red cell distribution width
ROC: Receiver operating characteristic
TC: Total cholesterol
TG: Triglycerides
VLDLs: Very low-density lipoproteins
